# Preparation and Characterization of Hydrophilic Polymer Based Sustained-Release Matrix Tablets of a High Dose Hydrophobic Drug

**DOI:** 10.3390/polym14101985

**Published:** 2022-05-13

**Authors:** Niaz Ali Khan, Amjad Khan, Rooh Ullah, Majeed Ullah, Amal Alotaibi, Riaz Ullah, Adnan Haider

**Affiliations:** 1 Department of Pharmacy, Abasyn University, Peshawar 25000, Pakistan; niaz68826@gmail.com (N.A.K.); dr.roohullah2012@gmail.com (R.U.); 2 Department of Pharmacy, Kohat University of Science and Technology (KUST), Kohat 26000, Pakistan; majeedkhattak@yahoo.com; 3 Department of Basic Science, College of Medicine, Princess Nourah bint Abdulrahman University, Riyadh 12211, Saudi Arabia; amaalotaibi@pnu.edu.sa; 4 Medicinal, Aromatic and Poisonous Plants Research Center, Department of Pharmacognosy, College of Pharmacy, King Saud University, Riyadh 12211, Saudi Arabia; rullah@ksu.edu.sa; 5 Department of Biological Sciences, National University of Medical Sciences, Rawalpindi 4600, Pakistan; adnan.haider@numspak.edu.pk

**Keywords:** clarithromycin, hydroxypropyl methyl cellulose, polymeric matrix

## Abstract

The objective of this study was the preparation and characterization of a sustained-release matrix tablet containing a high-dose hydrophobic drug and its comparison with marketed products. In the present study, HPMC was applied as the matrix-forming polymer for the sustained release of clarithromycin (500 mg). The compatibility of clarithromycin and excipients was studied using a binary mixture approach and compatible excipients were selected. Matrix tablets were prepared using the high-shear wet granulation technique. Tablets were compressed using oblong (19 mm), shallow concave punches, under a compression weight of 900 mg/tablet. The flow of granules was evaluated by determining their bulk density, tapped density, angle of repose, Hausner ratio, and Car’s index. Compressed tablets were tested for their physical parameters, mechanical characteristics, drug content, and in vitro drug release, as per United States Pharmacopeia (USP). Clarithromycin is a drug having poor water solubility and showed compatibility with all the excipients used in the formulation of polymeric matrix tablets. FTIR spectra of clarithromycin, before and after being subjected to the stress conditions, confirmed the compatibility of clarithromycin and other ingredients of the matrix tablets. All the formulations exhibited good rheological characteristics and all the parameters related to flow showed results in the acceptable range. Physically, matrix tablets were smooth and shiny, without any surface defects. Weight variation (±5%) and drug content of the tablets (95–102%) were within the pharmacopeial limits. HPMC successfully sustained the drug release for 24 h. It is concluded from the study that dissolution rate of clarithromycin can be sustained using hydrophilic polymer (HPMC) as a release-controlling agent.

## 1. Introduction

Despite their wide acceptance, higher dose frequency is the main limitation of oral solid dosage forms [1], resulting in missed doses and variation in drug concentration in blood. This phenomenon is to be critically considered in the case of drugs with a narrow therapeutic window and in conditions where the drug concentration in blood is required to be consistent. In conventional dosage forms, the drug is immediately released after oral administration, is absorbed from the gastrointestinal tract (GIT), and reaches the general circulation. The drug produces its response after achieving a therapeutic concentration in blood, and is metabolized and eliminated from the body [2,3]. The drug response peaks when its concentration in blood is high, and thereafter gradually declines to a sub-therapeutic level. To provide a longer-term effect, the drug concentration needs to be maintained at a certain level for a longer period of time. 

During the past few decades, a number of strategies have been adopted by the pharmaceutical scientist to maintain therapeutic drug concentration in blood. Among these, the most simple and robust method is to sustain the drug release to maintain a constant drug concentration in blood for a longer period. The sustained-release dosage form remains in the GIT and releases the drug at the rate equal to its elimination rate, thereby maintaining the required amount of the drug in blood for a long time. The main objective of designing a sustained-release dosage form is to achieve a therapeutic drug concentration in blood, maintain it for longer time, and overcome its fluctuation [4]. Some of the benefits of sustained-release formulations are:

- Reduced dose frequency, leading to no chance of a missed dose and subsequent enhancement in the patients’ compliance [5];
- Fluctuations in concentration of the drug in blood are overcome and a uniform level is maintained [6];
- Potent drugs can be administered in a safe way by controlling their release [5].

Polymers (large molecules made up of many small repeating units) have a variety of applications in pharmaceuticals, and their importance is steadily increasing. The characteristics of polymers are dependent on their molecular weight, chain length, and branching [7]. On the basis of solubility, polymers may be water soluble or soluble in organic solvents. 

Among hydrophilic polymers, hydroxypropyl methylcellulose (HPMC) has been widely used in the formulation of dosage forms for different purposes, such as a binder, monolithic matrix, and release modifier [8], due to its compatibility, better safety profile, and easy availability [9,10]. HPMC is a derivative of cellulose and forms a swollen gel upon contact with water. Water penetration into the core of the matrix and subsequent drug release are controlled by the swollen gel layer. Release of the drug from the HPMC matrix is based on:

- Diffusion;
- Erosion;
- Mixed release (combination of diffusion and erosion).

The dissolution rate of the drug from polymeric matrices is governed by physico-chemical characteristics of the active pharmaceutical ingredient (API), and the nature and quantity of the excipients included in the formulation. Generally, the process of diffusion is observed during the release of hydrophilic drugs. Upon penetration of water, the matrix becomes swollen, and the drug is dissolved and diffuses out of the matrix [11] at a pre-determined rate. In the case of hydrophobic drugs, the drug release occurs after erosion of the matrix. Similarly, water-soluble excipients present in matrix tablets dissolve earlier and micro-channels are developed, which increase water penetration into the core of the matrix and increase the dissolution rate. A number of studies have reported an increase in the dissolution rate by increasing the quantity of lactose (a water-soluble excipient) in the matrix tablets [12], and a decrease in the dissolution rate with the inclusion of di-basic calcium phosphate (a water-insoluble excipient) [13]. Changes in the GIT conditions can also affect the mechanism of the dissolution rate due to enzymatic activity and variation in pH of the medium from highly acidic to alkaline. Alcohol has a solubilizing effect on HPMC, and the Food and Drug Administration (FDA) has issued a warning about dose dumping due to rapid drug release from the matrix upon alcohol consumption. 

**Figure 1:**
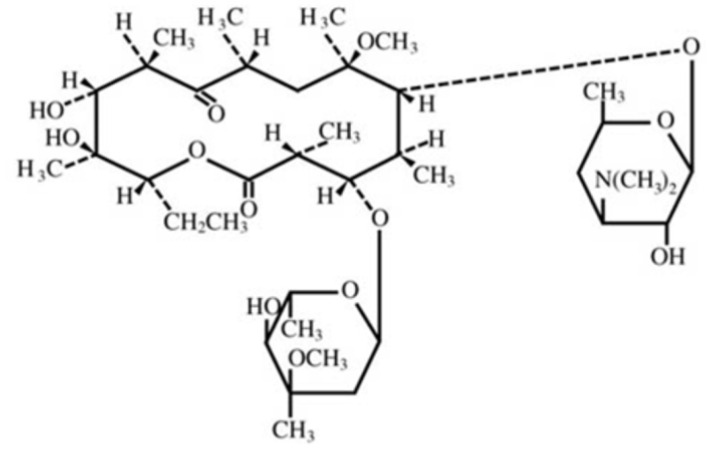
Schematic presentation of structural formula of clarithromycin.

Clarithromycin is an erythromycin derivative (6-*O*-methylerythromycin) macrolide [14]. The molecular formula of clarithromycin is C_38_H_69_NO_13_, with corresponding molecular weight equal to 747.96 g/mol. Physically, clarithromycin is a white or off-white powder with a crystalline nature. It is insoluble in water and can be solubilized in different organic solvents such as ethanol, acetone, acetonitrile, and methanol [15]. The structural formula of clarithromycin is presented in Figure 1. 

Clarithromycin is prescribed twice or three times per day, depending upon the severity of the infection. During antibiotic therapy, a consistent blood concentration of the drug is desired, whereas divided doses lead to a variation in blood plasma concentration. There is a need for a controlled-release dosage form of clarithromycin that can avoid the problems of multiple dosing and provide a consistent drug concentration in blood. The current study aimed to control the drug release of clarithromycin after oral administration via formulation as polymeric matrix tablets. Different formulations were developed using HPMC as a release-controlling polymer and evaluated for quality control parameters as per the official compendia. Dissolution profiles of the developed formulations were compared with conventional marketed products using a model-independent approach. 

## 2. Material and Methods

### 2.1. Material and Instrumentation

Clarithromycin (99.95% pure with respect to the USP standard; Wuxi Hexia Chemica Company, Wuxi, China) was the model drug, and excipients included lactose (Kerry company, Raunheim, Germany), HPMC (Dow Chemical Company, Wiesbaden, Germany), magnesium stearate (Linghu xingwing Chemical Co., Ltd., Huzhou, China) and talc. Different instruments, such as a rotary compression machine (Yenchen, Taiwan), digital tablet testing apparatus (PharmaTest, Hainburg, Germany), dissolution testing apparatus (Pharma Test, Hainburg, Germany), friabilator (Pharma Test, Hainburg, Germany), super mixing granulator (Yenchen, Taiwan), IR spectrophotometer (FTIR Prestige, Shimadzu, Japan), and HPLC (Perkin-Elmer, Langenfeld, Germany), were used in the preparation and evaluation of polymeric matrix tablets.

### 2.2. Evaluation of Compatibility of Drug and Polymer

The compatibility of clarithromycin with HPMC and other excipients was evaluated on the basis of a drug excipient compatibility study. Samples were prepared using the binary mixture approach [16], subjected to stress conditions, and checked for physical consistency, clarithromycin content, and FTIR spectra. Samples were prepared as per Table 1, by physical mixture, using 2 g of each material. 

Samples were prepared as per Table 1 and packed in air-tight glass containers. Samples were stored at stress conditions (40 ± 2 °C and 75 ± 5% R.H.) for 90 days and checked at regular intervals, i.e., at days 1, 30, 60, and 90.

The physical consistency (color and physical appearance) of each sample was evaluated by visual inspection. The drug content in each drug-containing sample was determined by HPLC. An FTIR spectrophotometer (Shimadzu, Japan) was used for recording the IR spectra of each sample using a KBr disc. A small quantity of the sample was mixed with KBr and crushed to a fine powder. A mixture of the sample and KBr was loaded to the sample folder with gentle tapping and spectra were recorded at 4000–400 cm^−1^ in transmittance mode. Data were processed using I.R. Solutions software, version 1.10. 

### 2.3. Preparation of Clarithromycin Matrix Tablets

Matrix tablets of clarithromycin were prepared via high-shear wet granulation using HPMC as the matrix-forming polymer. The main steps of the process were mixing all of the ingredients (drug, polymer, and other excipients) in a dry state; wet massing with the binder; and drying, sizing, lubrication, and compression of granules. All the ingredients, as listed in Table 2, were weighed accurately, sifted through a mesh having a 600 µm pore, and loaded into a lab-scale high-shear wet granulator (Yenchen, Taiwan). All the ingredients were mixed in a dried state by rotating the main impeller at 150 rpm, while the speed of the chopper was 2200 rpm. Water was used as the binder and was added at the rate of 1 L/20 s. Wet granulation was performed at high speeds (150 and 2500 rpm) of the main impeller and chopper. Drying of wet granules was achieved in a fluidized bed drier by a stream of hot air. Initially, simple air was passed for one minute and then the temperature of the inlet air was increased to 80 °C. Sizing of the dried granules was carried out using a cone mill having a rotating mesh of 2 mm pore, revolving at 800 rpm. Granules were lubricated and compressed using a rotary compression machine. The compression machine was fitted with oblong shallow concave punches (19 mm), with an engraved “*f*” on one side. The compression weight of tablets was 900 mg/tablet and, for each formulation, a minimum of 250 tablets was prepared. In the formulation of sustained-release clarithromycin tablets, lactose monohydrate was used as a diluent to make up the bulk. This was added in different quantities in different formulations to make the final weight of tablet equal to 900 mg/tablet. Talcum was used as an anti-adherent and magnesium stearate was used as a lubricant.

### 2.4. Pre Compression Evaluation

Granules for all the formulations were tested for their flow parameters as per USP [17]. Bulk density and tapped density of granules were determined by placing a weighed quantity of granules in a graduated cylinder [17]. For determination of the tapped volume, known quantity of granules was placed in a cylinder and tapped manually. Reduction in the volume of granules was noted after each 100 taps, until the volume became constant; this volume was noted as the tapped volume. The weight and tapped volume of the granules were used for the calculation of the tapped density. The mean values of the bulk and tapped density were used to calculate Car’s Index and the Hausner ratio [17] using Equations (1) and (2), respectively.

(1) $${\mathrm{Car}}^{\prime }s\mathrm{Index}=\frac{\mathrm{Dc}-\mathrm{Da}}{\mathrm{Dc}}\times 100$$

(2) $$\mathrm{Hausner}\mathrm{Ratio}=\frac{\mathrm{Dc}}{\mathrm{Da}}$$

where “Dc” and “Da” denote the tapped and bulk density of the granules, respectively. 

A glass funnel was used for determination of the angle of repose of the granules, as per USP [17]. The glass funnel was fitted to a stand at the height of 5 cm from the table top and its lower opening was closed with a cotton plug. Granules were placed in the funnel and allowed to flow by removing the cotton plug. The radius and height of the powder heap was measured and the angle of repose was calculated using the following equation:

(3) $$\propto =\tan^{-1}\left(\frac{H}{r}\right)$$

In Equation (3), α denotes the angle of repose, and “H” and “r” are the height and radius of the granule heap, respectively. 

### 2.5. Evaluation of Matrix Tablets 

#### 2.5.1. Physical Parameters of Tablets

Physically, the matrix tablets of clarithromycin were checked for appearance and surface characteristics. The thickness and diameter of randomly selected tablets (*n* = 10) were measured with a digital hardness and thickness tester (Pharma Test, Hainburg, Germany), and their average values were calculated. For calculation of weight variation, tablets were randomly selected and weighed individually, and average weight was calculated. The difference in the individual weight of tablets and the average weight were used for the calculation of the percent weight variation using Equation (4) [17]:

(4) $$Percent\;weight\;variation=\frac{Individual\;weight-Average\;weight}{Average\;weight}\times 100$$

#### 2.5.2. Mechanical Strength of Tablets

The mechanical strength of matrix tablets of clarithromycin was evaluated by determining their crushing strength, specific crushing strength, and tensile strength, and friability testing, as per USP [17]. The crushing strength of tablets (*n* = 10) was measured via digital tablet hardness, and the specific crushing strength and tensile strength were calculated using the mean values of the crushing strength and the thickness of tablets, as per USP, using the following equations:

(5) $$\mathrm{Tensile}\mathrm{Strength}=\frac{2F}{\pi \mathrm{DH}}$$

and

(6) $$\mathrm{Specific}\;\mathrm{Crushing}\mathrm{Strength}=\frac{F}{\mathrm{HD}}$$

where “F” denotes the crushing strength of the matrix tablets, “D” is the diameter, and “H” denotes its thickness; π is the constant of proportionality.

For determination of friability, tablets (*n* = 10) were randomly selected, dedusted, and subjected to 100 rotations in a Roche friabilator at 25 rpm [17]. When rotations completed, tablets were evaluated for physical defects (breakage, chipping, capping, and lamination) and reweighed, and percent weight loss was calculated as follows:

(7) $$Percent\;weight\;loss=\frac{Wb-Wa}{Wb}\times 100$$

where:

*Wb* = weight of tablets before subjecting to friability

*Wa* = weight of tablets after friability testing.

#### 2.5.3. Determination of Drug Content

The amount of clarithromycin in the sustained-release matrix tablets was determined as per USP [17]. Matrix tablets were taken and crushed, and powder containing about 2000 mg of clarithromycin was placed in a flask (capacity = 500 mL). Methanol (350 mL) was added to the flask and shaken for 30 min. Further methanol was added to the solution to make up the volume. An aliquot (3 mL) of this solution was diluted to 100 mL by adding a mobile phase and analyzed by HPLC. A mixture of methanol and mono-basic potassium phosphate (0.067 M) in 65:35 by volume was used as the mobile phase. The stationary phase consisted of a 4.6 mm × 150 mm column with L1 packing and was eluted with the mobile phase at the rate of 1 mL/min. The temperature of the column oven was kept at 50 °C and the detection wavelength was 210 nm. For the preparation of a standard solution, a weighed quantity of clarithromycin (250 mg) was dissolved in a sufficient quantity of methanol to obtain a concentration of 625 µg/mL. An aliquot (10 mL) of the solution was placed in a volumetric flask of 50 mL capacity and the volume was made up with the mobile phase. The final concentration of the standard solution was 125 µg/mL. Analysis of the standard and sample solutions was carried out by HPLC, and their peak areas were compared to obtain the percent concentration of clarithromycin in the sample preparation.

#### 2.5.4. Determination of Dissolution Rate 

The dissolution rate of the controlled-release clarithromycin tablets was studied in 900 mL of 0.1M acetate buffer (pH 4.6) [17]. One tablet was placed in each flask containing dissolution media at 37 ± 2 °C. USP dissolution apparatus II (peddle) was used at 50 rpm for stirring the dissolution media. Samples were collected at specified time intervals and analyzed for drug content using HPLC. Details of chromatographic conditions and the mobile phase are mentioned in the Section “Determination of drug content”. The volume of the dissolution media was corrected with fresh dissolution media after each sampling. 

#### 2.5.5. Comparison of Dissolution Profiles

A model-independent approach was used for comparing dissolution profiles of matrix tablets with each other and with conventional tablets of clarithromycin. The model-independent approach is based on the similarity factor (*f*_2_), dissimilarity factor (*f*_1_) and dissolution efficiency (D.E.). The dissimilarity factor (*f*_1_) and similarity factor (*f*_2_) were calculated by Equations (8) and (9), respectively [18,19,20]:

(8) $$f_{1}=\frac{\sum \left[R_{t}-T_{t}\right]}{\sum R_{t}}\times 100$$

(9) $$f_{2}=50\times \log\{[1\times (1/n)\sum {\left(R_{t}-T_{t}\right)}^{2}{]}^{-0.5}\times 100\}$$

In Equations (8) and (9) “*R_t_*” and “*T_t_*” denote the dissolution rate of the standard and tested product at time “*t*”, respectively. Similarity of the two dissolution profiles was ensured by a value of “*f*_2_*”* equal to or greater than 50.

In addition to the mentioned parameters, time taken by each formulation to release 50% (T_50%_) and 100% (T_100%_) of the drug was also determined. Similarly, the quantity of drug released at different time points was also compared. 

## 3. Results and Discussion

### 3.1. Selection of Excipients

For the preparation of polymeric matrix tablets, compatibility with clarithromycin and with other excipients was used as a basis for selection of the excipients. Compatibility of clarithromycin and other excipients was evaluated by preparing samples (physical mixture), subjecting these samples to stress conditions (40 ± 2 °C and 75 ± 5% relative humidity), and evaluated the samples at specified time intervals. Results showed that clarithromycin was compatible with polymer (HPMC) and other excipients. Incompatibility between drug and excipients leads to instability of dosage form and reduced bioavailability. Both physical and chemical interactions among ingredients (API and excipients) of the formulation can lead to incompatibility and subsequent instability of the product. 

Both types of interactions can be evaluated by analysis of the physical state and drug content of the sample. Furthermore, compatibility can be confirmed by the IR analysis as changes in functional groups can be clearly evaluated from the IR spectra. In the current study, all the samples were evaluated for physical consistency, drug content (where applicable) by HPLC, and IR spectra. The results presented in Table 3 show that all the samples remained stable for a specified period of time under stress conditions. The physical state of the samples remained the same before and after being subjected to stress conditions (40 ± 2 °C and 75 ± 5% relative humidity). The drug content of the samples remained unaffected and no extra peak was observed during HPLC analysis. Similarly, changes were not observed in the IR spectra of all the samples. These results indicate that clarithromycin is compatible with polymer and other excipients used in the preparation of polymeric matrix tablets. 

**Figure 2:**
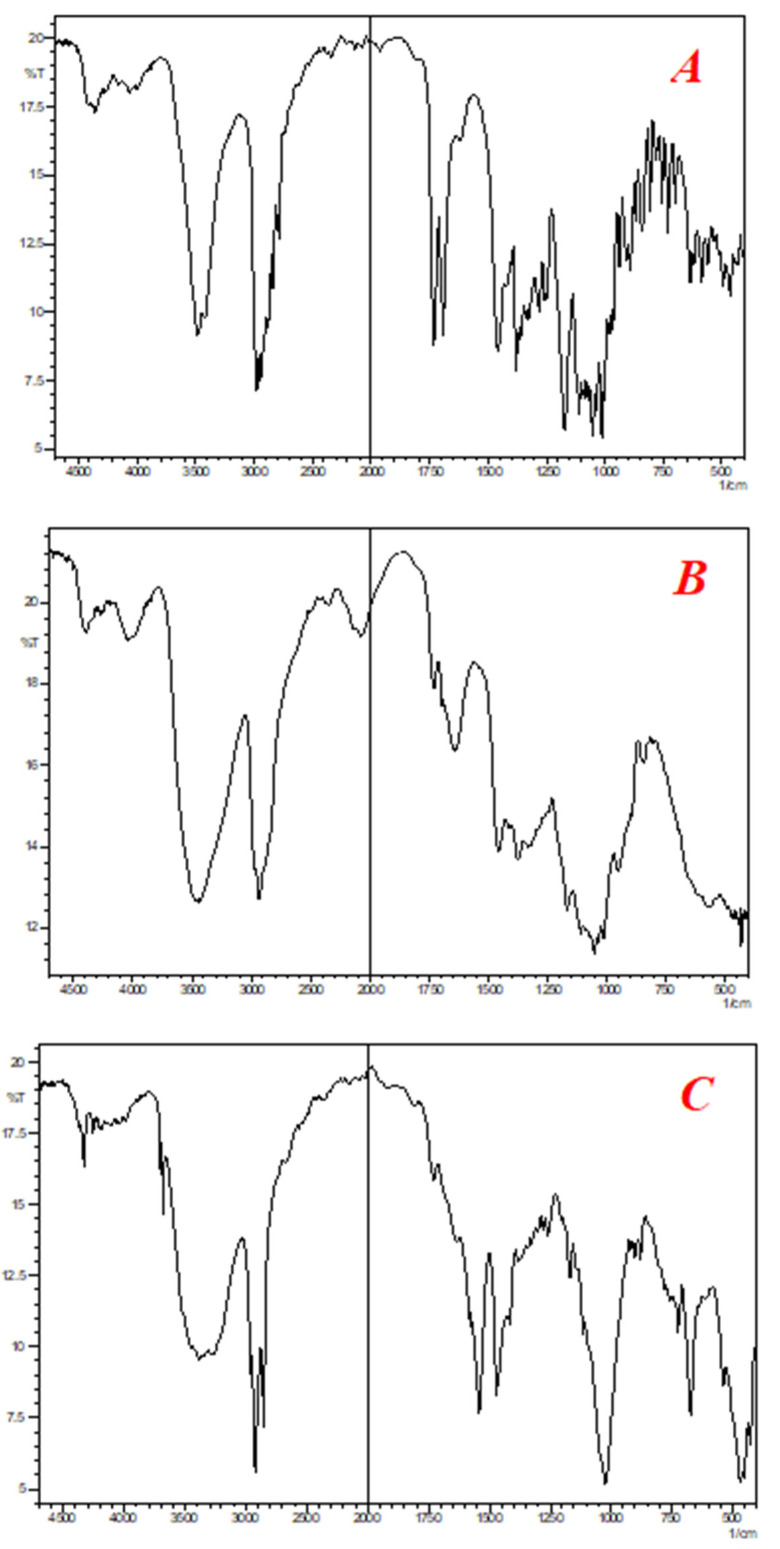
FTIR Spectra of (**A**) clarithromycin, (**B**) HPMC, and (**C**) sustained-release matrix tablets of clarithromycin.

FTIR spectra of clarithromycin, excipients, and mixture of clarithromycin and excipients are presented in Figure 2.

### 3.2. Pre-Compression Evaluation

The high-shear wet granulation technique was applied for preparation of controlled released polymeric matrix tablets of clarithromycin. Before compression, granules were evaluated for different parameters related to their flow, and results are compiled in Table 4. A larger quantity of polymeric material usually results in harder granules, with better flow and compressibility. As HPMC was used as the drug-release-controlling polymer in three concentrations (10, 15, and 20%, *w*/*w*), granules for all the formulations were freely flowing. The angle of repose for all the formulations was in the range of 20 to 23, indicating better flow. Similarly, the results of Car’s index and Hausner ratio also showed better flow characteristics of the granules, as shown in Table 4. The flow of the granules improved with the increase in the quantity of polymer. The best results were obtained with Formulation-3, which had 20% polymer. A higher quantity of polymer results in hard granules with a uniform structure, which leads to better flow [17].

### 3.3. Post-Compression Evaluation

After compression, tablets from all the formulations were tested for physical parameters, mechanical strength, drug content, and dissolution rate. 

#### Physical Parameters of Matrix Tablets

Matrix tablets of clarithromycin were compressed by shallow concave, oblong punches with a 19 mm diameter. The surface of the tablets was smooth and shiny, and tablets were free of sticking and picking. A shiny surface and lack of defects are an indication of proper lubrication of the granules. The matrix tablets were compressed at 900 mg/tablet and the variation in their weight was less than ±1%, as shown in Table 5. Better flow of the granules resulted in low weight variation of the tablets. The thickness of the tablets was in the range of 6.4–6.7 mm. The drug content of the tablets was determined by HPLC and was found to be within the official limits (100 ± 10%).

### 3.4. Mechanical Strength of Tablets 

The mechanical strength of the matrix tablets was estimated on the basis of crushing strength (hardness), specific crushing strength, tensile strength, and friability, determined as per USP. The crushing strength of the tablets was in the range of 18–24 kg and increased with the increase in the quantity of polymer in the formulation. The highest crushing strength was observed with Formulation-3, which contained 20% of HPMC. It has been reported that HPMC has a strong binding action and keeps the particle in close contact [6]. The increase in the quantity of HPMC led to more binding action and formation of stronger granules, resulting in the higher crushing strength of the tablets. The specific crushing strength and tensile strength of the tablets also showed similar results, as shown in Table 5, indicating their better strength. The friability of that matrix tablets was determined as per USP. After friability testing, the tablets were free of any physical deformation (breaking, capping, lamination, and chipping) and weight loss during the test was within the official limits (>1%).

### 3.5. Drug Release from Polymeric Matrix Tablets

**Figure 3:**
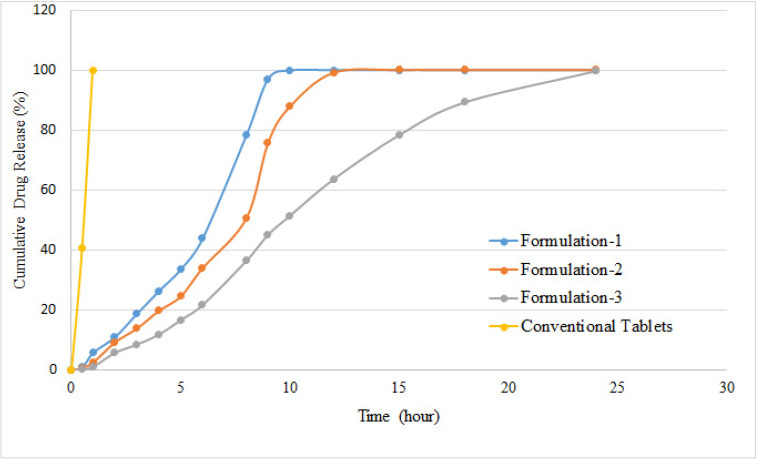
In vitro drug release from controlled-release tablets and conventional tablets of clarithromycin.

The objective of the study was to sustain the release of clarithromycin using HPMC as a matrix-forming polymer. HPMC is a hydrophilic polymer and forms a gel layer upon water contact [21,22]. The dissolution medium passes through the gel layer, dissolves the drug, and diffuses it in a controlled manner. The dissolution rate is controlled by the thickness of the gel layer, diffusion through the gel layer, and the gel layer’s erosion. Furthermore, penetration of water causes a decrease in the glass transition temperature to 37 °C and elongation of polymer chains, which facilitates drug release. When dissolution media interacts with an HPMC matrix, it passes through different stages. Initially, the glass transition temperature of the polymeric matrix is decreased to 37 °C, with very little movement of the macromolecules. The decreased movement of molecules in the matrix results in a lower rate of diffusion of dissolution media into the inner core of the matrix. Disruption of hydrogen bonds in polymer chain takes place by penetration of dissolution media, which causes swelling of the matrix and, ultimately, its erosion. The drug is dissolved by the penetrating dissolution media and diffuses from the swollen matrix. The rate of drug diffusion is controlled by the drug’s solubility in dissolution media, the micro-environment of the matrix, and the thickness of the diffusion layer [12]. Furthermore, drug release increases with the increase in the rate of erosion of the polymeric matrix. In a gel-forming polymeric matrix, diffusion is the dominating rate-controlling mechanism, whereas in a non-swelling polymer, drug release is controlled by polymer erosion. In the case of polymers that have higher viscosity, a stable gel layer is formed upon contact with water and polymer erosion is very low. The dissolution rate of the drug is controlled by its diffusion following Fick’s law of diffusion, with a square root time dependency [22]. As HPMC is a gel-forming polymer and swells upon contact with dissolution media, drug release was diffusion dependent and decreased with the increase in the quantity of polymer. At a lower concentration (10% *w*/*w*), drug release was sustained for 10 h, whereas at a higher concentration (30% *w*/*w*), drug release was sustained for 24 h at a slower rate. The rate of drug release was uniform with a higher concentration of HPMC and almost zero-order kinetics were followed, as shown in Figure 3. At lower concentrations of HPMC, drug release was slower initially and an abrupt release was observed at a later stage of dissolution, which may be due to the mixed effect of diffusion and erosion. 

### 3.6. Comparison of Dissolution Profiles

**Figure 4:**
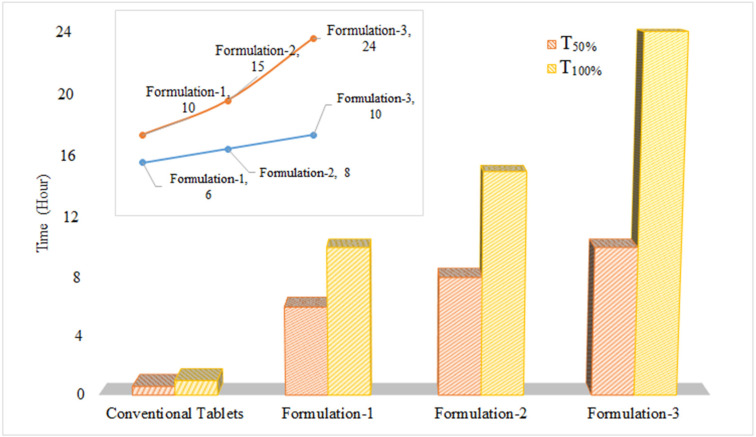
Comparison of time taken for 50% (T_50%_) and 100% (T_100%_) drug release from polymeric matrix tablets and conventional tablets of clarithromycin.

A comparison of dissolution profiles was undertaken with conventional tablets of clarithromycin (500 mg) using a model-independent approach based on dissimilarity and similarity factors. Furthermore, other dissolution parameters, such as the amount of drug released at different time intervals (Q_2h_, Q_5h_, Q_12h_, and Q_24h_) and time taken for 50% and 100% of drug release, were also evaluated. In conventional tablets, complete drug release was observed within 60 min. A marked decrease in the dissolution rate was observed following the inclusion of HPMC in the formulation. At a lower concentration of HPMC, drug release was slower than that of conventional tablets, whereas it was faster than that of the formulations containing larger quantities (Formulations-2 and -3). Comparative evaluation showed a linear relationship between the quantity of HPMC and the increase in time for 50 and 100% drug release from the matrix, as shown in Figure 4. 

**Figure 5:**
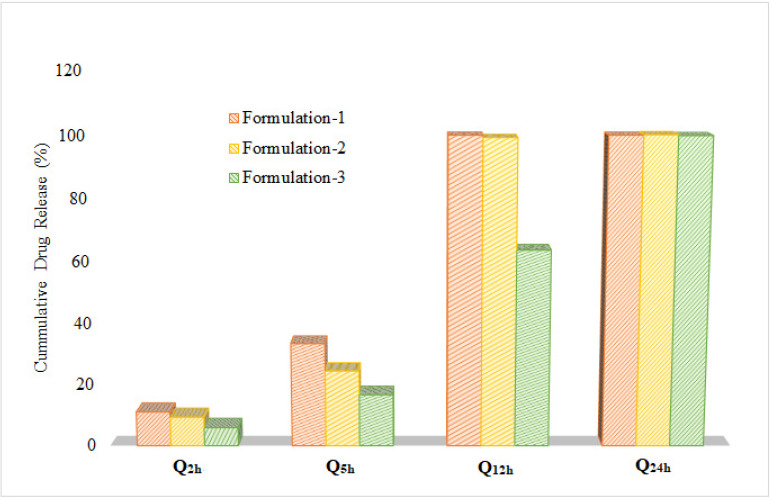
Comparison of quantity of drug release from polymeric matrix tablets of clarithromycin at different time intervals.

Similarly, an increase in the concentration of HPMC reduced the drug release at different time points, as shown in Figure 5. 

The values of the dissimilarity factor were above 50 for all the dissolution profiles, indicating their significant difference. The highest value of *f*_1_ was observed in the comparison of conventional tablets and Formulation-3, indicating that HPMC significantly modified the dissolution rate. Similarly, the values of the similarity factor were very low, as shown in Table 6. All the dissolution profiles were different from each other and showed no similarity. 

## 4. Conclusions

The objective of the study was the preparation and characterization of a sustained-release matrix tablet containing a high-dose hydrophobic drug and its comparison with marketed products. The release of clarithromycin was sustained up to 2 h due to the formulation of hydrophilic polymeric matrix tablets using the high-shear wet granulation method. The drug excipient compatibility study showed that all the selected excipients were compatible with the drug and polymer. All the developed formulations showed better flow, compressibility, and mechanical strength. The developed formulations complied with pharmacopeial quality control standards for assay and dissolution testing. The dissolution rate of clarithromycin was sustained for 24 h at a consistent rate. It is concluded from the study that hydrophilic polymers can sustain the release of a high-dose and hydrophobic drug for longer period of time, which will decrease dose frequency and the fluctuation in the drug concentration in blood. 

## 5. Future Prospect of the Study

The presented manuscript covers the formulation and development of polymeric matrix tablets of clarithromycin and their in vitro evaluation. In future, in vivo evaluation of the developed formulations can be carried out.

## Figures and Tables

**Table 1:** Composition of samples used in drug excipient compatibility test.

Sample	Composition
Sample-1	Pure clarithromycin
Sample-2	Clarithromycin + HPMC in 1:1 by weight
Sample-3	Mixture of clarithromycin, HPMC and all the excipients in 1:1 by weight

**Table 2:** Composition of sustained-release clarithromycin matrix tablets.

Ingredients	Formulation-1	Formulation-2	Formulation-3
Clarithromycin	55.56	55.56	55.56
HPMC	10.00	15.00	20.00
Lactose Monohydrate	29.94	24.94	19.94
Talcum	3.00	3.00	3.00
Mg. stearate	1.50	1.50	1.50
Purified water	QS	QS	QS

Quantities are given as % *w*/*w*; Mg. Stearate: magnesium stearate; Q.S.: quantity sufficient.

**Table 3:** Results of drug excipient compatibility study.

Analysis Time	Characteristic	Sample-1	Sample-2	Sample-3	Sample-4
Before subjecting to stress conditions	Drug Content	* 100.03 ± 0.38	_	99.69 ± 0.73	99.81 ± 0.49
	IR Spectra	† Complied	Complied	Complied	Complied
	Physical State	Complied	Complied	Complied	Complied
After subjecting to stress conditions	Drug Content	100.01 ± 0.93	_	99.81 ± 0.70	99.96 ± 0.37
	IR Spectra	Complied	Complied	Complied	Complied
	Physical State	Complied	Complied	Complied	Complied

*: Results are presented as mean ± SD (*n* = 3); †: The term complied meant that IR spectra remained unaffected.

**Table 4:** Results of pre-compression evaluation of controlled-release tablets of clarithromycin.

Properties (Unit)	Formulation-1	Formulation-2	Formulation-3
Angle of repose (°)	23.19 ± 0.05	20.93 ± 0.05	20.56 ± 0.03
Bulk density (g/mL)	0.292 ± 0.04	0.301 ± 0.02	0.312 ± 0.02
Taped density (g/mL)	0.339 ± 0.03	0.352 ± 0.05	0.362 ± 0.04
Car’s Index (%)	15.55 ± 0.04	15.73 ± 0.02	16.44 ± 0.03
Hausner Ratio	1.16 ± 0.03	1.17 ± 0.06	1.16 ± 0.05

**Table 5:** Post-compression evaluation of polymeric matrix tablets.

Status	Parameters (Unit)	Formulation-1	Formulation-2	Formulation-3
Physical Parameters	Weight (mg)	910	917	909
	Weight variation (%)	0.98	1.06	1.19
	Diameter (mm)	19	18.98	19.05
	Thickness (mm)	6.49	6.66	6.53
	Drug content (%)	1.16	1.04	1.02
Mechanical strength	Hardness (Kg)	22	18	23.5
	Specific crushing strength (kg/Nmm^2^)	0.056	0.045	0.06
	Tensile strength (kg/Nmm^2^)	0.113	0.0907	0.12
	Friability (%)	0.14	0.21	0.16

**Table 6:** Values of the similarity factor (*f*_2_) of conventional tablets and controlled-release tablets of clarithromycin.

Products	Conventional Tablets	Formulation-1	Formulation-2	Formulation-3
Conventional Tablets	100	13	11	6
Formulation-1	13	100	29	17
Formulation-2	11	29	100	43
Formulation-3	6	17	43	100

## Data Availability

All the data is presented in the manuscript without any restrictions.
